# Development of standardized nursing terminology for the process documentation of patients with chronic kidney disease

**DOI:** 10.3389/fnut.2024.1324606

**Published:** 2024-02-01

**Authors:** Shi Pu, Hongmei Peng, Yang Li, Xia Huang, Yu Shi, Caiping Song

**Affiliations:** ^1^Department of Nephrology, The Key Laboratory for the Prevention and Treatment of Chronic Kidney Disease of Chongqing, Chongqing Clinical Research Center of Kidney and Urology Diseases, The Second Affiliated Hospital, Army Medical University (Third Military Medical University), Chongqing, China; ^2^President Office, The Second Affiliated Hospital of Army Medical University (Third Military Medical University), Chongqing, China

**Keywords:** chronic kidney disease, nutritional management, standardized nursing terminology, European Nursing care Pathways, subset

## Abstract

**Introduction:**

European Nursing care Pathways (ENP) is a professional care language that utilizes software to map care processes and utilize the data for research purposes, process control, and personnel requirement calculations. However, there is a lack of internationally developed terminology systems and subset specifically designed for the nutritional management of CKD. The aim of this study was to create a subset of the standardized nursing terminology for nutrition management in patients with chronic kidney disease (CKD).

**Materials and methods:**

According to the guidelines for subset development, four research steps were carried out: (i) Translation of version 3.2 of the ENP (chapter on kidney diseases) and understanding of the framework structure and coding rules of the ENP; (ii) Identification of relevant six-dimensional nursing terms; (iii) Creation of a framework for the subset; (iv) Review and validation by experts.

**Results:**

A subset for CKD nutritional care was created as part of this project, comprising 630 terms, with 17 causal relationships related to nursing diagnoses, 115 symptoms, 31 causes, 34 goals/outcomes, 420 intervention specifications and 13 resources, including newly developed care terms. All terms within the subset have been created using a six-step maintenance procedure and a clinical standard pathway for nutrition management in the SAPIM mode.

**Implications for nursing practice:**

This terminology subset can facilitate standardized care reports in CKD nutrition management, which is used to standardize nursing practice, quantify nursing, services, guidance on care decisions, promoting the exchange and use of CKD nutrition data and serve as a reference for the creation of standardized subset of nursing terminology in China.

## 1 Introduction

Accurate and meaningful documentation of patient care is crucial for ensuring quality and patient security. The electronic nursing record (ENR) uses the nursing staff’s workstation to record medical records Information, use of computer information networks to collect and record clinical nursing information data. This approach aims to increase the efficiency of care and improve the overall quality of care (1). The content is the performance of care services and the original recording of the patient’s condition changes. With the continuous development of electronic medical records, the degree of digitization and the structure of the medical records have improved considerably. However, the description was mainly entered in unstructured free text, without the possibility of a logical review and assessment. This represents a risk and hinders the improvement of the quality of care (2, 3). Standardized formulations for the presentation of care processes can prove decisive for future outcome measurements.

Standardized nursing terminologies (SNTs) are standardized specialist languages that are rooted in nursing practice and science. They provide a uniform standard for the recording and documentation of care data and form the basics of nursing care information systems (4). SNTs play an important role in evaluating the effectiveness of care practice, help administrators to make appropriate decisions, promote communication between healthcare providers and promote the development of practical knowledge (5). The American nurse association recognizes 7 terminologies or classifications that are widely used in nursing practice (6). However, domestic researchers compared the content of clinical nursing practice with the standardized terminology. They found that the existing terminology is very extensive and contains numerous terminologies (7), and there was a lack of disease-specific classifications (8) for mapping a patient with chronic renal failure. This presents nursing staff with the challenge of quickly identifying the necessary nursing diagnoses and interventions and hinders the application and development of the terminology system in China (9). Therefore, the development of a subset of specialized terminology based on home care practice in the context of nutrition management for people with chronic kidney disease are recommended.

As the nursing classification European Nursing care Pathways (ENP) is best able to map the nursing diagnoses of a person with chronic kidney disease, it was decided to use ENP as the starting point for the research project and to make the research findings available for further development. ENP has been developed in Germany since 1998 and is published in several languages by RECOM GmbH (Thieme Group) in Germany and Europe as book publications, online applications and databases for integration into software products (10, 11). The latest ENP 3.2 version was released in 2021. This system offers several advantages, including an extensive collection of terminology and detailed descriptions, high accuracy, specialization-based classification, ease of use, mapping and compatibility in various care environments (12). It comprises a total of 520 nursing diagnoses and over 6,000 associated interventions. Each ENP practice guideline is aligned with the current international literature and is particularly suitable for electronic data evaluation, e.g., for outcome measurements. An ENP practice guideline consists of six terminology modules (nursing diagnoses with characteristics, causes, goals/outcomes, resources, intervention concepts and detailed intervention descriptions), which are the special framework structure of ENP that was used in the research work.

Chronic kidney disease (CKD) has become a major global public health problem (13). Malnutrition is a common complication of CKD and a contributing factor to disease progression (14), cardiovascular events, and mortality (15). As a result, nutritional management has become an essential part of treatment strategy for patients with CKD and offers potential intervention options to improve the disease prognosis (16). However, there is currently no standardized terminology for nutritional care in CKD. As a result, practices such as diagnoses, interventions and outcomes cannot be captured consistently in EHRs using generally recognized standards. This restriction dramatically hampers quality improvement of CKD management.

This project is a joint project that combines home care practice with the terminology development guidelines of the International Organization for Standardization. It builds on of the framework structure and the terminology system of the ENP, a subset of the standardized care system terminology related to nutrition in chronic kidney disease.

## 2 Materials and methods

In August 2022, the exclusive translation, development and application license for ENP version 3.2 was granted, and the ENP practice guidelines relevant to the mapping of nutritional problems in patients with CKD will be released.

A systematic approach was used to collect nursing terminology from the literature. A total of 19 articles were published on the development of a standardized classification system for nursing diagnoses were included in this study. The literature is reviewed based on the topic of the article, the method used in the study etc., to understand and focus on the current development and use of the term subset on the specific methods and steps of subset development. The results show that the most important research methods used included the literature review method, the content-based analysis method, theoretical research method, Delphi method and mapping method. At the same time, combined with the terminology subset development guidelines issued by official organizations, the development and evaluation process was performed in four phases between June 2022 and September 2023: (i) Translation of version 3.2 of the ENP practice guidelines by understand the framework structure and coding rules of the ENP; (ii) Identification of relevant six-dimensional nursing terms; (iii) Creation of a framework for the subset; (iv) Review and validation by experts.

### 2.1 Translation of selected ENP practice guidelines for a person with CKD

There is no standard translation method for the translation of care terminology. According to the Brislin translation standard (17), the German version of ENP (chapter on kidney diseases) 3.2 translated, and three steps were applied: (i) double translation, (ii) back-translation and (iii) re-examination. The purpose of the translation was to master the ENP framework and coding rules and understand the content of existing articles and the coding ID for nutritional management in renal disease chapter to facilitate localization and expansion. Therefore, no experts were included in this study validation and cultural debugging of the translated version.

### 2.2 Identify relevant terms of six concept groups

Two primary data sources were used to identify the content for the subset/enhancement of ENP for CKD nutrition management. The first was a full literature review, including research papers, published guidelines and selected nursing/nutrition textbooks. The second was the actual clinical nursing data that was collected by nurses while they provide nutritional management.

Search using all identified keywords and index terms was undertaken across 9 Chinese and English databases, including PubMed, CINAHL, Embase, Cochrane Library, CNKI, Sinomed, Wanfangand VIP. Collected literatures, clinical practice guidelines and expert consensus on nutrition in patients with CKD from January 2000 to June 2022 (Although research on nutrition in patients with CKD began to appear in 1988, we considered the research at that time to be too old. And the National Kidney Disease Foundation of the United States issued the first version of the Kidney Disease Quality Improvement Program (KDOQI) practice guidelines for nutritional therapy in 2000 (18). Since then, a large number of studies related to nutritional therapy have been accumulated). Combination of subject words and free words, and searched all synonyms as far as possible, the search words include chronic kidney disease/renal disease, nutrition/nutritional/diet, therapy/management, nursing/care, diagnose, intervention, outcome, etc.

Evidence was found and evaluated for important clinical issues such as the assessment and monitoring of nutrition in CKD patients, the nutrition treatment programs for different stages of CKD, nutrition programs for diabetics and non-diabetics CKD patients and the recommended nutrient intake. At the same time, the CKD nutritional therapy guidelines issued by authoritative institutions and the care terminologies in authoritative medical nursing and nutrition textbooks were evaluated to determine the content and the scope of the nursing terminology subset for nutrition management in CKD.

In addition to the literature, the second data source was the electronic patient file of a chronic kidney disease facility Management Center, the second affiliated hospital of the Army Medical University. The center adopted a patient-centered and nurse-led chronic management model, which offers long-terms nutrition management services for more than 15,000 people over 10 years. Through the self-developed CKD nutrition management information system, patients’ files were established, disease assessment, nutrition assessment, nutrition analysis, individualized nutrition plan, recipe formulation, nutrition education for patients, and effect evaluation, etc. The system is seamlessly connected with the outpatient doctor workstation of the hospital Information system (HIS), electronic medical record (EMR), and laboratory information system (LIS). The basic information of patients, laboratory tests, auxiliary tests, medication, hospitalization records and other data can be automatically extracted. And docking with the patient APP terminal, to provide patients with timely and effective whole-process case information management from hospitalization to discharge and home.

### 2.3 Creating a conceptual framework for the subset

As different diseases have their own best models of care, it is necessary to select scientific theory as the theoretical framework according to the characteristics of specialties of diseases and to construct a standardized nursing terminology system (19). This study decided to develop a subset for process documentation of patients with CKD based on the framework structure of ENP. At the same time, standardized nutrition management model (SAPIM model) (20) was used to organize the terms of intervention specifications, which is the concept group with the most terms.

### 2.4 Verification and validation by experts

In this study, the newly developed terms were reviewed in advance using the Delphi method (21). We selected experts the German center of RECOM GmbH (Thieme Group) and the leading hospitals in China. The experts’ criteria were as follows: (i) those working in clinical nursing or nursing management in the department of nephrology for more than 10 years; (ii) Master’s degree or higher; (iii) familiar with CKD Nutrition Management or ENP; (iv) with the title of Nurse Manager or higher. The content of the ENP framework and purpose of the development of subsets were explained in detail to each expert; they were asked to comment on the wording, expression, completeness, feasibility, scientific nature and rationality of the first draft of the terminology database.

The questionnaire consisted of three parts. (i) Description of the questionnaire; (ii) Basic information questionnaire for experts; (iii) Content subset framework of standardized care terms for CKD nutrition management. Two rounds of questionnaires were distributed on site distribution and email. The experts were asked to provide feedback within 2 weeks and a reminder was sent out at the end of the first week. The second round of questionnaires was based on the screening results of the first round and expert opinions.

## 3 Results

### 3.1 Translation of selected ENP practice guidelines for a person with CKD

In the ENP Version 3.2, an ENP nursing diagnosis relating to nutrition reads “The patient is undernourished people due to persistent energy/nutrient deficiency (ID 555),” in which a total of 451 items such as characteristics, causes, interventions, etc. are offered to represent the patient situation. For people with CKD, a total of 65 of these conceptual terms were confirmed in our data analysis to be with domestic clinical nursing. This can be explained by the fact that the nursing diagnosis also covers other causes of malnutrition and is not only focused on people with CKD. With the data-based review of the ENP practice guideline, a higher level of evidence can be reported for this practice guideline in the future. On this basis, this study expanded the localized and species-specific terms.

### 3.2 Drafting a standardized nursing terminology for CKD nutrition issues

A retrospective review of electronic patient records examined the data routinely documented by full-time nurses CKD management services in the Electronic Center for Chronic Kidney Disease Management Database. 1,593 data records for patients with CKD who were hospitalized from January to December 2022 were reviewed. At the same time, 16 guidelines for CKD nutrition management were published by authoritative institutions and 4 authoritative nursing textbooks were evaluated. It was that the terminology used to calculate the protein and energy requirements in different stages of the disease, liquid and inorganic salts, electrolytes, vitamins, metabolic acidosis, exogenous nutrient supplementation, etc. On the basis of a targeted literature review and a retrospective examination of nutritional care documentation in EHRs, totaling 14 unique care settings diagnoses, associated symptoms (*n* = 101), causes (*n* = 76) and goals/outcomes (*n* = 34) were determined, care measures (*n* = 23), intervention specifications (*n* = 382) and resources (*n* = 11), they were also significantly from the data sources.

The research team combined professional judgment in the terminology of specific care interventions, and hospital management system and, after discussion, added 7 more care measures, such as the implementation of cooking courses, the use of the portion model for food demonstrate the distribution of meals and analyze the type of fat consumed, etc.

### 3.3 The conceptual framework of the subset

**Figure 1:**
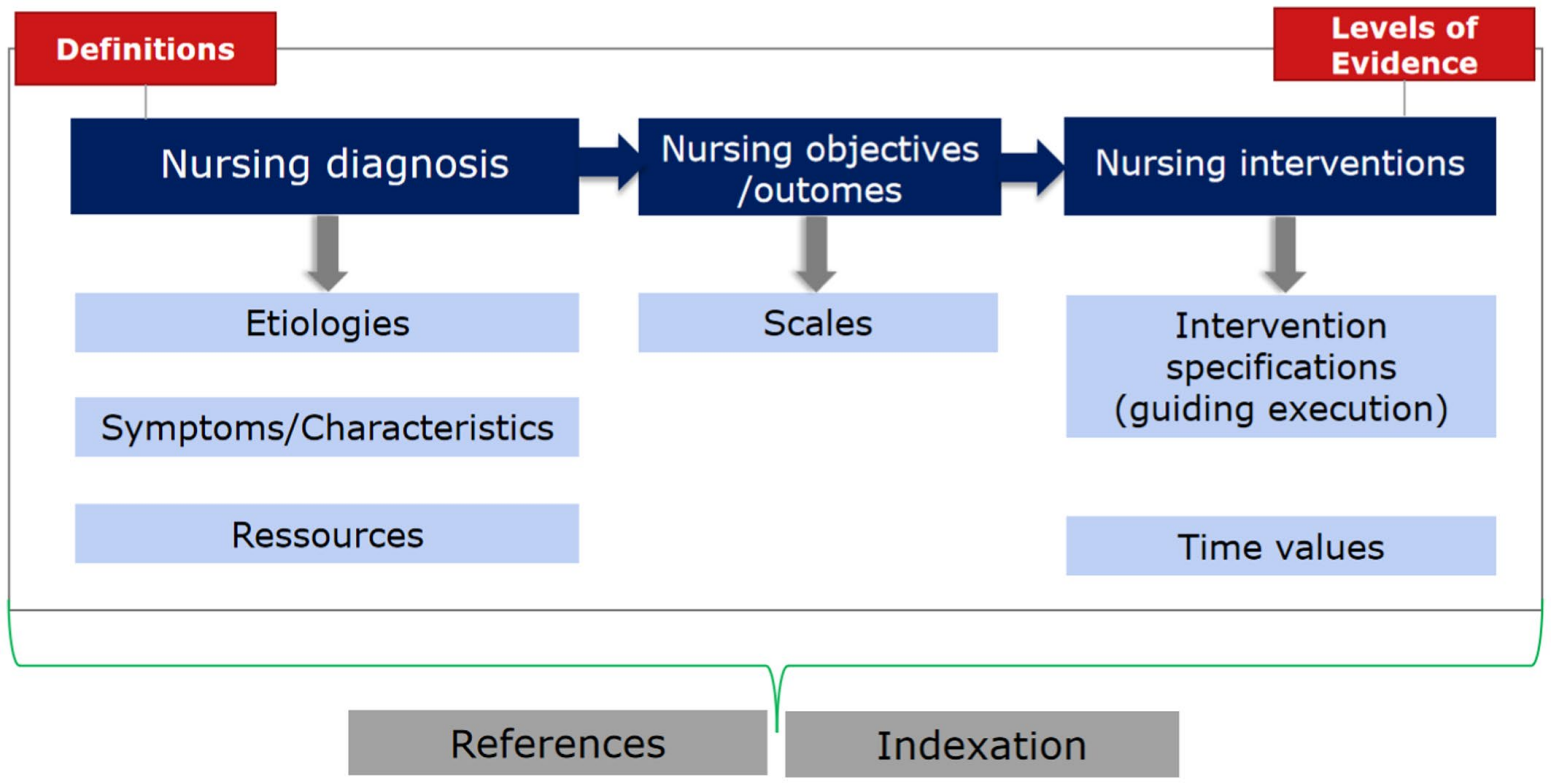
Structure of ENP-practice guidelines.

**Figure 2:**
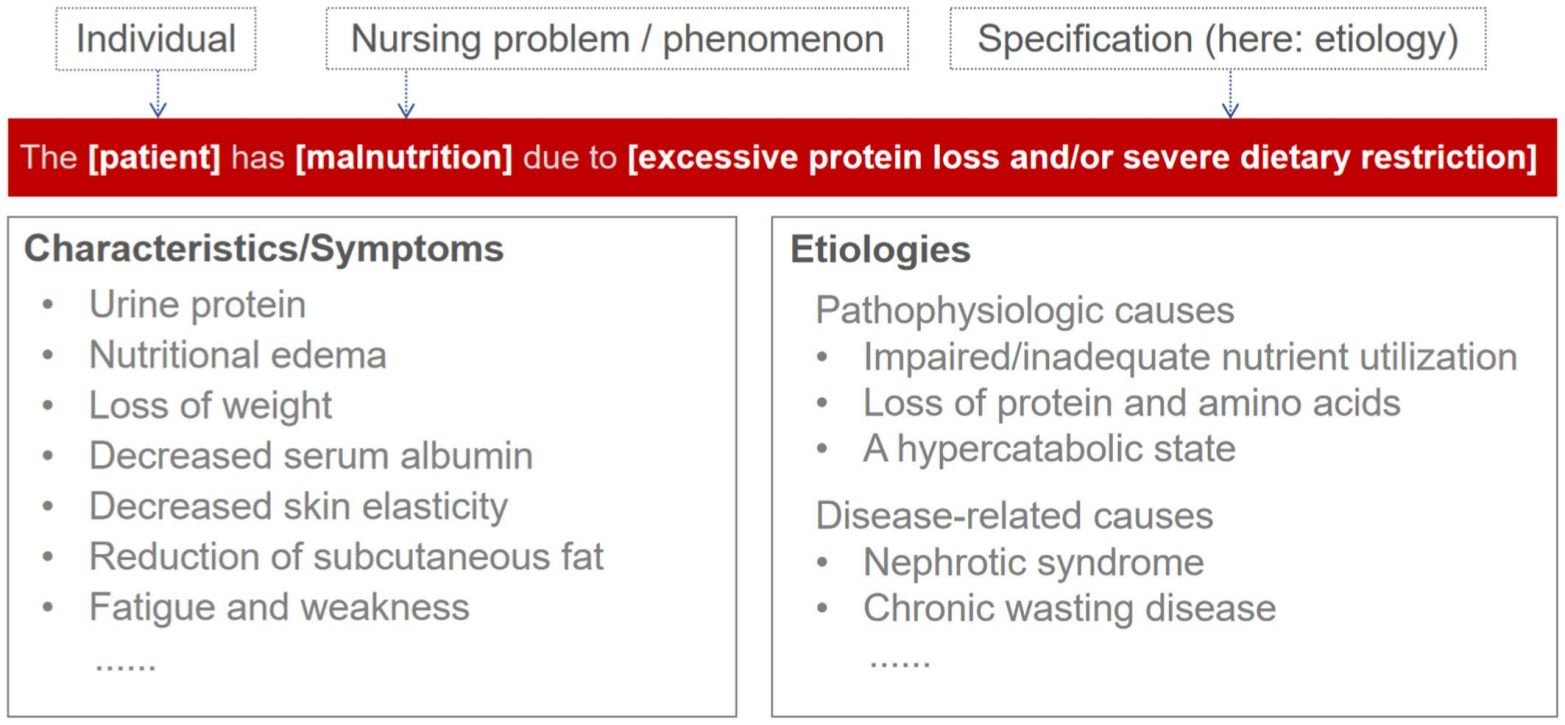
Reference points of the nursing diagnoses in ENP.

ENP as the practice guidelines developed from the pre-combination and the nursing classification which offer nurses professional support to illustrate the nursing care process by using standardized formulations, such as nursing diagnoses, characteristics, etiologies, resources, nursing outcomes, and interventions, see Figure 1. ENP nursing diagnoses in general represent a systematic clinical assessment of a care recipient’s reactions to current and/or potential health problems and/or life processes. Nursing diagnoses are therefore part of the nursing process and form the basis for the selection of nursing measures that are used to achieve the nursing goals developed together with the person being cared for. The ENP nursing diagnosis usually comprises the nursing problem and a specification of (etiologies or symptoms) and thus precombinatorially forms a nursing diagnosis, see Figure 2. Each identified term should be evaluated against the level of evidence of the ENP version 3.0 guidelines for nursing diagnoses and practice.

**Figure 3:**
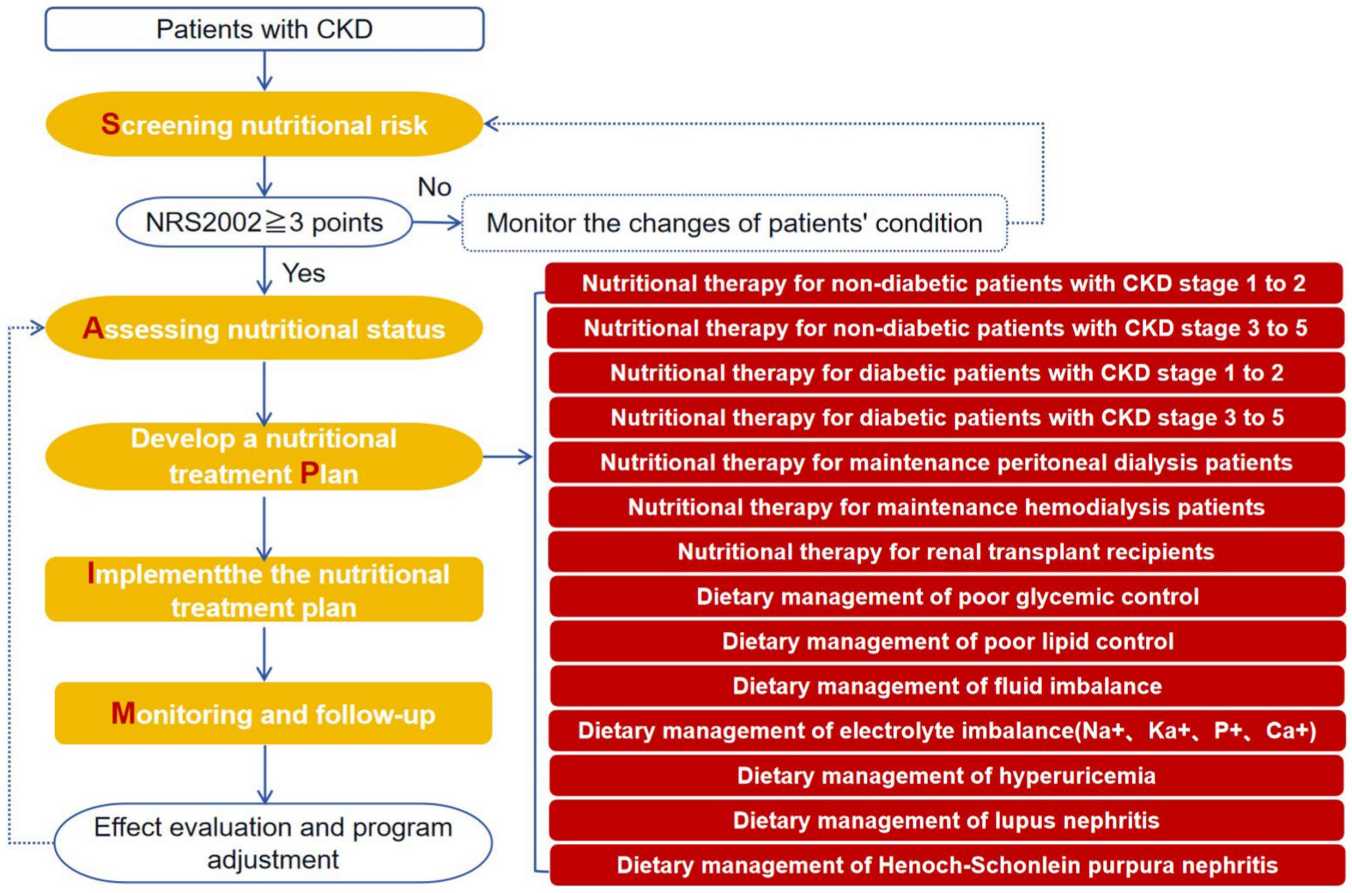
Standard clinical pathway for nutritional management in CKD based on the SAPIM model (five-step method).

The principles of standardized nutritional management in CKD are: under the guidance of evidence-based on medical guidelines and standards, using clinical logic and habits, identifying patients with recognize malnutrition or nutritional risks as early as possible and formulate individual, healthy support, treatment plan, careful monitoring of complications and effects of supportive nutritional therapy, and dynamic adjustment of the treatment plan for nutritional support with timely feedback, see Figure 3.

### 3.4 Finalization of a standardized nursing terminology for nutrition management in CKD

#### 3.4.1 Review and validation by experts

Subsequently, a total of 24 questionnaires were distributed in the 2 rounds of this study. The positive coefficients of experts in the first and second rounds both were 100%, indicating a high degree of participation and importance in this study. The Kendall coordination coefficient W of two rounds were 0.132 and 0.156, respectively. The coordination coefficient of each dimension was between 0.201 and 0.273 (*p* < 0.05), indicating that the expert scores were consistent. The coefficient of variation for the importance of each item in the two rounds were 0 ~ 0.32 and 0 ~ 0.25, indicating that the experts agreed on the content of the index. The coefficient Cr value of expert authority degree was 0.9125, indicating high reliability of expert scoring and authoritative and reliable research results.

In the first round of consultations, some experts pointed out that nutrition-related terms relevant to nursing such as hemodialysis, peritoneal dialysis and kidney transplant recipients should be added. Two experts mentions that the term mainly refers to oral feeding, and if protein and energy requirements are not after a nutritional intervention and oral nutritional supplements, tube feeds or parenteral nutrition are met, should recommended. Finally, 35 terms were added such as “Explain the importance of diet to the patient/caregiver,” “Guide the proper use of food quantification AIDS,” “Calculated peritoneal glucose absorption,” “Demonstration of the operation of four-square meal method” and “Assess the degree of edema and scope” in the concept group of nursing intervention; “Reduce the frequency of diet-related lupus activity,” “Reduce the frequency of diet-related allergic reactions” and “Improve the quality of life of patients” were added in the concept group of nursing outcome; One expert suggested incorporating “Digital health literacy” into the concept group of resource. At the same time, the experts focused on the deletion or amendment of terminology that to combine rarely used and similar terms.

In the second round of consultations, 6 terms such as “Instructing patients/caregivers to use the Protein-based Food Exchange Checklist,” “ask patient to record glucose data while at home” and “Educating patients about fat types (saturated, unsaturated, trans),” and some items were revised and improved.

#### 3.4.2 Update of intervention terminologies based on clinical questions

According to the clinical question “How does a healthy diet affect clinical outcomes in patients with CKD?,” using the systematic review method to prove that “healthy dietary patterns to reduce the incidence of mortality, ESKD and CVD in patients with CKD.” Add terminology entry for interventions: “Encourage patients to follow a Mediterranean diet or DASH diet.”

According to the clinical question “Is a very low protein diet safe and effective?” using the systematic review method to prove that “compared to a conventional low-protein diet, a very low-protein diet delay the progression of kidney disease without increasing the overall risk of death and malnutrition events and can be safely used in patients with stage 3–5 chronic kidney disease.” Add terminology entry for interventions “a very low protein diet (0.3 g per kilogram per day) with keto acid supplementation.”

According to the clinical question “Is it possible to assess nutrition-related symptoms from a patient’s perspective?” We have developed and validated the CKD Patient-Reported Outcome Scale (CKD-PROs). Add terminology entry for interventions: “Use CKD-PROs to assess nutritional status.”

After further induction and merging, a subset for CKD nutritional care with 630 related terms were identified, the retained 65 terms from the ENP practice guidelines for a person with CKD were included. The items identified as necessary for people with CKD to map the patient case in an electronic patient record will be incorporated into the further development of the ENP, see Table 1.

**Table 1:** Content framework for standardized nursing terminology in connection with nutrition in patients with chronic kidney disease.

Patient conditions, causal relationships which require differentiated nutritional management in CKD (*n* = 17)	
1. Non-diabetic patients with CKD1-2 have protein-energy wasting due to excessive protein loss and/or strict dietary restriction	
2. Non-diabetic patients with CKD stage 3–5 have protein-energy wasting due to excessive protein loss and/or strict dietary restriction	
3. Patients with CKD stage 1–2 diabetes have protein-energy wasting due to excessive protein loss and/or strict dietary restriction	
4. Patients with CKD stage 3–5 diabetes have protein-energy wasting due to excessive protein loss and/or strict dietary restriction	
5. Patients on maintenance hemodialysis dialysis have protein-energy wasting due to nutrient clearance by dialysis and/or strict dietary restriction	
6. Patients on maintenance peritoneal dialysis have protein-energy wasting due to nutrient clearance by dialysis and/or strict dietary restriction	
7. Renal transplant recipients have protein-energy wasting due to increased protein/energy wasting	
8. The patient have fluid overload due to hypoproteinemia and/or water and sodium retention	
9. CKD patients with diabetes mellitus have elevated blood glucose levels due to abnormal glucose metabolism/unreasonable diet/drug side effects	
10. The patient have elevated lipid levels due to abnormal renal function and/or unreasonable diet	
11. The patient have elevated uric acid levels due to abnormal renal function and/or unreasonable diet	
12. The patient have elevated serum potassium levels due to abnormal renal function and/or unreasonable diet	
13. The patient have calcium and phosphorus metabolism disorder due to abnormal renal function and/or unreasonable diet	
14. The patient have elevated blood sodium levels due to abnormal renal function and/or unreasonable diet	
15. The patient developed metabolic acidosis due to abnormal renal function	
16. The patient lacked special knowledge of lupus nephritis diet	
17. The patients lacked knowledge of a special diet for Henoch-Schonlein purpura nephritis	
Symptoms (*n* = 115)	
Degree of decline in glomerular filtration rate (*n* = 6)	Clinical diagnosis (*n* = 27)
Abnormal biochemical markers (*n* = 26)	Clinical manifestation (*n* = 41)
Anthropometric data (*n* = 8)	Inadequate dietary intake (*n* = 2)
Etiologies (*n* = 31)	
Causes related to kidney disease	Other disease-related causes
Physiological cause	Dietary Reasons
Patient’s causes	Family reasons
Social support related reasons	Economic reasons
Educational level is limited	Genetic factor
Unhealthy lifestyle	Drug-related causes (*n* = 8)
Fluid intake was more significant than output	Excessive water restriction
Intravenous potassium supplementation was excessive	Metabolic acidosis
Causes of hormone Secretion	Negative emotional impact
Abnormal immune response	Disorder of acid–base balance
Contact with allergenic objects	Taking cause allergic food
Disorder of acid–base balance	Increased dietary acid load
Resources (*n* = 13)	
Participate actively in the decision-making process	Good compliance
Understand the need for dietary interventions	Receiving support from family caregivers
The ability to accept and overcome illness	Show perseverance to acquire new skills
Willing to learn new things	Self-efficacy
Understand the need for dietary interventions	Can tolerate diet therapy/nursing intervention
Set goals and try to achieve them	Family resilience
Digital health literacy	
Results of care (*n* = 34)	
Improve malnutrition	The intake of each nutrient was accurately measured
The intake of nutrients met the requirements	Improve patients’ disease self-management ability
Reducing the risk of complications	Identify the causes of malnutrition
Improve dietetic habit	Accept and follow an established dietary plan
Cultural habits are recognized	Master nutrition-related knowledge (*n* = 10)
Use eating AIDS effectively	Slowing disease progression
Nutrition-related indicators were well-controlled (*n* = 7)	The symptom is reduced edema
Maintain fluid balance	Return to normal weight
Improve the quality of life	Reduce the frequency of diet-related lupus activity
Reducing the frequency of diet-related allergic reactions	
Maintenance measures (*n* = 420)	
Screening for nutritional risk (*n* = 7)	Assessing nutritional status (*n* = 60)
Develop a nutritional treatment plan (*n* = 1)	Implement the nutritional treatment plan (*n* = 278)
Nutrition monitoring and follow-up (*n* = 72)	Others (*n* = 2)

## 4 Discussion

According to the evaluation standard for electronic medical records, which is published by the National Institutes of Health Commission of the People’s Republic of China, the core elements and functions of the current nursing information system should include: (i) the implementation of the care process as a work process; (ii) with international framework for nursing terminology; (iii) introduction of a knowledge base for nursing. In this way, the data collection of the entire care process can be realized. Data analysis and evaluation can then be such as measurement of workload, analysis of care status, performance management in care, quality improvement in care, evidence-based evidence development, etc. (22). It is therefore necessary thoroughly study the methodology of international terminology formation, choose a research path that are suitable for local practice, and a standardized terminology system for care with Chinese Characteristics. On the basis of the comprehensive consideration of systematics, completeness and operability, this study decided to build the nutritional care Big Data of CKD on ENP.

Nutritional management is an essential part of the whole process of treating CKD, which is of great importance for improving diagnosis and treatment, delaying progression and improving the quality of life of patients and the prognosis of CKD. However, CKD is a complex disease state with many pathological types. It is influenced by complications, hospitalization, replacement therapies and other factors, so that the nutritional management strategy for CKD is not just a restrictive diet (23). Nurses play a crucial role in providing appropriate nutritional support for CKD patients, from screening and assessment on admission to the management of patients’ food intake in clinical practice work, preparation and implementation of nutrition plans, ensuring nutrition delivery and prevention and management of nutrition-related complications (24). Information systems for care are generally in a state of semi-informedness and lack of structured design. In view of the huge amounts of data that arise from the long-term comprehensive care of a large CKD population, as there is no standardized care language, there is a lack of uniform normative terminology and formats for care reports, which has a direct impact on the secondary use of care data and facilitates the exchange of information and the sharing of data in different areas, diseases and languages. The lack of resources for information management has become the “bottleneck” of the administration; at the same time, it restricts the implementation and promotion of nutritional management in CKD, resulting in a minimized number of patients who comprehensive and high-quality management.

In the current classification system of nursing terminology, there are only a few terminologies that relate to nutrition care, such as “Using a feeding tube” and “Rinsing or washing the feeding tube” in the CCC system, “Management of nutritional status” and “Monitoring of nutrition” in the ICNP system. Firstly, these terminologies can be used for the nutritional management of all patients with chronic wasting diseases and specific terms for specific diseases are missing. For example, the critical points of nutritional management for CKD patients mainly include the intake and control of energy, protein, sodium, potassium and calcium, phosphorus and fluid, instead of enteral or parenteral nutrition. With ENP, the mapping of a person with CKD was still the most accurate and complete compared to the other systems. Nevertheless, this work has shown that specific aspects of differentiated nutritional management are missing. Secondly, the existing nutritional and related care terms are too broad to ignore certain content, and do not distinguish between the various degree of the same condition. Various measurement indicators and nutritional indicators are used in daily care work. Assessment tools should be used rationally for screening, assessment and dynamic monitoring of nutritional status of CKD patients. Adequate energy and micronutrient intake programs should be recommended patients depending on the stage of the disease and the assessment results (25). Finally, the development of precision medicine, artificial intelligence and big data, research in the field of precision nutrition, nutritional epidemiology, dietary patterns or nutrient selection, metabolic surgery and nutrition administration is also making progress (26). However, the existing terminology system has not been updated according to the latest theories and findings; examples are intermittent fasting, the Mediterranean diet and scales with patient information to assess nutrition-related symptoms. Although there are broader nutrition-related terms in the subset of stroke patients developed by the ENP. The content focuses on the treatment of dysphagia and tube feeding. Nutrition-related care intervention for CKD places more emphasis on health education and improves patients’ self-confidence management skills. For example, the terms “exchange portions based on dietary protein” are commonly used foods can be divided into categories according to their sources and properties. Similar foods contain similar proteins and energy within a certain weight, which can guide patients to exchange foods of the same kind and enrich the range of foods on offer.

In the present study, the subset developed by us can be used as a standardized recording instrument directly in CKD nutrition management care documents so that the documents are clear, easy to understand and reduce the workload of nursing staff. As a set of coded terminologies, it can be compatible with other medical terminology systems in the world that have been mapped with ENP, and the descriptive structure of practice guidelines results in ENP having a significantly lower level of abstraction and a higher level of granularity compared to other classification systems. The items developed in this work will be incorporated into the ENP development of version 3.4 and can thus close the identified gaps in the future. With the help of a specific technology platform, caregivers involved in CKD management in different countries, and identify and understand regions using appropriate codes. The standardized and coded care data are easy to store, extract, transfer and use from the computer to exchange and share information, promote the development of nursing research on CKD nutrition management. As a dynamic language system, as nursing practice and research evolve, the subset is continually revised based on the latest theory and evidence to ensure that they correspond to actual needs and the latest findings. In the field of computers, it is has created a computer language that can be recognized. In information science, the standardized terminology system can carry out information dissemination smoothly and fulfill the purpose of the exchange of information (27).

## 5 Conclusion

In this study, a new subset of care terms was developed according to the development requirements of international terminology and will be included in the future ENP version 3.4. The research work makes a significant contribution to promoting the prevalence of nursing classifications and interoperability. It fills the gap in standardized care therapy systems in CKD nutrition management. The research results were made available to the ENP developers in order to check which elements would be included in the standardized terminology. In the following study, we will apply the constructed terminology system to the target group as far as possible, identify the obstacles to the application of theory in practice and solve them to recognize the value of standardized nursing terminology. This study provides nephrology nurses and all those providing professional care for CKD with a systematic approach to identify nursing diagnoses/outcomes and interventions that are geared toward individualized and humane care.

## Data availability statement

The original contributions presented in the study are included in the article/supplementary material, further inquiries can be directed to the corresponding authors.

## Ethics statement

The studies involving humans were approved by the Institutional Review Board of The Second Affiliated Hospital, Army Medical University (2018-No.006-01). The studies were conducted in accordance with the local legislation and institutional requirements. Written informed consent for participation was not required from the participants or the participants' legal guardians/next of kin in accordance with the national legislation and institutional requirements. Written informed consent was obtained from the individual(s) for the publication of any potentially identifiable images or data included in this article.

## Author contributions

SP: Conceptualization, Writing – original draft. HP: Data curation, Writing – original draft. YL: Data curation, Writing – original draft. XH: Data curation, Writing – original draft. YS: Conceptualization, Writing – original draft. CS: Conceptualization, Funding acquisition, Methodology, Supervision, Writing – review & editing.
